# CAG Expansions Are Genetically Stable and Form Nontoxic Aggregates in Cells Lacking Endogenous Polyglutamine Proteins

**DOI:** 10.1128/mBio.01367-16

**Published:** 2016-09-27

**Authors:** Ashley A. Zurawel, Ruth Kabeche, Sonja E. DiGregorio, Lin Deng, Kartikeya M. Menon, Hannah Opalko, Martin L. Duennwald, James B. Moseley, Surachai Supattapone

**Affiliations:** a Department of Biochemistry, Geisel School of Medicine at Dartmouth College, Hanover, New Hampshire, USA; b Department of Pathology, Schulich School of Medicine and Dentistry, University of Western Ontario, London, Ontario, Canada; c Department of Medicine, Geisel School of Medicine at Dartmouth College, Hanover, New Hampshire, USA

## Abstract

Proteins containing polyglutamine (polyQ) regions are found in almost all eukaryotes, albeit with various frequencies. In humans, proteins such as huntingtin (Htt) with abnormally expanded polyQ regions cause neurodegenerative diseases such as Huntington’s disease (HD). To study how the presence of endogenous polyQ aggregation modulates polyQ aggregation and toxicity, we expressed polyQ expanded Htt fragments (polyQ Htt) in *Schizosaccharomyces pombe*. In stark contrast to other unicellular fungi, such as *Saccharomyces cerevisiae*, *S. pombe* is uniquely devoid of proteins with more than 10 Q repeats. We found that polyQ Htt forms aggregates within *S. pombe* cells only with exceedingly long polyQ expansions. Surprisingly, despite the presence of polyQ Htt aggregates in both the cytoplasm and nucleus, no significant growth defect was observed in *S. pombe* cells. Further, PCR analysis showed that the repetitive polyQ-encoding DNA region remained constant following transformation and after multiple divisions in *S. pombe*, in contrast to the genetic instability of polyQ DNA sequences in other organisms. These results demonstrate that cells with a low content of polyQ or other aggregation-prone proteins can show a striking resilience with respect to polyQ toxicity and that genetic instability of repetitive DNA sequences may have played an important role in the evolutionary emergence and exclusion of polyQ expansion proteins in different organisms.

## IMPORTANCE

Polyglutamine (polyQ) proteins encoded by repetitive CAG DNA sequences serve a variety of normal biological functions. Yet some proteins with abnormally expanded polyQ regions cause neurodegeneration through unknown mechanisms. To study how distinct cellular environments modulate polyQ aggregation and toxicity, we expressed CAG-expanded huntingtin fragments in *Schizosaccharomyces pombe*. In stark contrast to many other eukaryotes, *S. pombe* is uniquely devoid of proteins containing long polyQ tracts. Our results show that *S. pombe* cells, despite their low content of endogenous polyQ proteins, exhibit striking and unexpected resilience with respect to polyQ toxicity and that genetic instability of repetitive DNA sequences may have played an important role in the emergence and expansion of polyQ domains in eukaryotic evolution.

## INTRODUCTION

In eukaryotes, proteins containing polyglutamine (polyQ) regions participate in a variety of normal biological functions, including cell cycle regulation (1), transcriptional regulation, and chromatin maintenance (2). However, overexpansion of the CAG repeat regions that encode polyQ domains within specific proteins can give rise to a group of inherited neurodegenerative diseases, known as polyQ expansion diseases (3). For example, Huntington’s disease (HD) occurs when the polyQ region of the huntingtin protein (Htt) contains more than 36 glutamine residues (4, 5).

PolyQ proteins are involved in many protein-protein interactions. These polyQ interactions may be mediated by their self-association in polar zipper structures and by coiled coil motifs (6, 7). This propensity of polyQ domains to self-associate may also play a key role in the pathogenesis of polyQ expansion diseases. A hallmark of such diseases is the presence of protein aggregates containing the pathogenic polyQ proteins. These aggregates have been speculated to be inherently cytotoxic as a consequence of sequestering other essential proteins (8, 9). The expanded polyQ region, in polyglutamine expansion diseases in particular, has been proposed to associate with other endogenous proteins containing glutamine tracts, such as CREB-binding protein, causing a loss of function phenotype (10–15). Furthermore, endogenous proteins with intermediate levels of polyQ expansion have been shown to influence host cell susceptibility to a misfolded pathogenic protein, as evidenced by the interaction of ataxin-2 containing intermediate-length polyQ repeats with the amyotrophic lateral sclerosis (ALS)-causative protein TDP-43, itself containing a prion-rich domain (16–18). Yet many studies have challenged this view and have suggested that polyQ aggregates can be benign or even offer protection from the toxicity associated with smaller, oligomeric conformers (19–25).

The taxonomical distribution of polyQ proteins provides unique insights into the possible role of normal (i.e., non-disease-associated) polyQ proteins. This distribution also provides intriguing opportunities to study polyQ misfolding, aggregation, and toxicity in living cells with distinct proteomic environments. Some organisms, such as *Drosophila melanogaster* and *Dictyostelium discoideum*, have evolved to carry a high number of these polyQ proteins. For example, 10.5% of the proteins expressed in *D. discoideum* contain polyQ domains (defined as a stretch of 10 or more consecutive glutamine residues) compared to 0.34% in humans. Recent work characterizing the effects of expanded polyQ Htt expression within *D. discoideum* (26) revealed that these cells effectively prevented pathogenic Htt aggregation and toxicity, except when stressed. These results suggest that the *D. discoideum* protein quality control system may have adapted to the presence of a large number of polyQ proteins, possibly by coevolving a highly active network of chaperone proteins.

Among yeast species in particular, there are striking differences in the abundance of polyQ proteins. A total of 79 proteins within the *Saccharomyces cerevisiae* genome contain polyQ regions (~1.1%), while the *Schizosaccharomyces pombe* proteome contains only three open reading frames with a 10-glutamine stretch (<0.1%), which is a uniquely low frequency among eukaryotes (2). Moreover, no proteins in *S. pombe* contain more than 10 polyQ proteins, which has been defined as the minimum length of a polyQ domain (2). Heterologous Htt exon 1 expression in *S. cerevisiae* is an established model for HD, characterized by Htt aggregation and cellular toxicity that is dependent on the length of the Htt polyQ region (27). This toxicity might stem from coaggregation of essential yeast polyQ proteins or, alternatively, from heterologous Htt aggregates physically disrupting essential processes. We reasoned that the near-absence of polyQ proteins in the *S. pombe* proteome might provide a unique opportunity to distinguish between these possibilities. Further, the *S. pombe* protein quality control system presumably evolved under minimal selective pressure to regulate polyQ aggregation, thereby generating a unique model to examine the relationship between protein quality control systems and polyQ protein aggregation.

Here, we expressed polyQ Htt proteins with polyQ expansion ranging from 25Q to 103Q in *S. pombe* cells*.* Our results document a striking resilience of *S. pombe* with respect to both polyQ aggregation and polyQ toxicity. We further observed unusual and unexpected genetic stability of the repetitive DNA sequences that encode polyQ expansion in *S. pombe*. These findings may help to elucidate how the benign and toxic functions of polyQ expansion proteins relate to the presence of endogenous polyQ proteins and their related protein quality control systems.

## RESULTS

### Only Htt with a greater length of polyQ expansion (103Q) aggregates in *S. pombe.*

We generated a series of constructs consisting of human huntingtin exon 1 with various lengths of CAG repeats, an amino-terminal FLAG epitope, and a C-terminal green fluorescent protein (GFP) tag (see Fig. S1 and Table S1 in the supplemental material). These constructs were integrated into the *S. pombe* genome and expressed by the inducible P3nmt1 promoter, which induces expression in the absence of thiamine. For comparison, a very similar construct using cyan fluorescent protein (CFP) in place of GFP was expressed in *S. cerevisiae* cells under the control of the galactose-inducible GAL1 promoter and a very similar construct using cyan fluorescent protein in place of GFP was expressed in *S. cerevisiae*. (Note that the longest construct expressed in *S. pombe* was Htt-103Q, whereas the longest construct expressed in *S. cerevisiae* was Htt-97Q, due to the genetic instability of the polyQ-encoding region in *S. cerevisiae* upon integration.) Upon induction in these systems, Htt protein was expressed in *S. cerevisiae* and *S. pombe* at the expected molecular mass as judged by Western blotting (see Fig. S2). The expression level of polyQ Htt with the strong P3nmt1 promoter in *S. pombe* was similar to that seen with the GAL1 promoter in *S. cerevisiae* (see Fig. S2; compare lanes 4 to 12).

**FIG 1:**
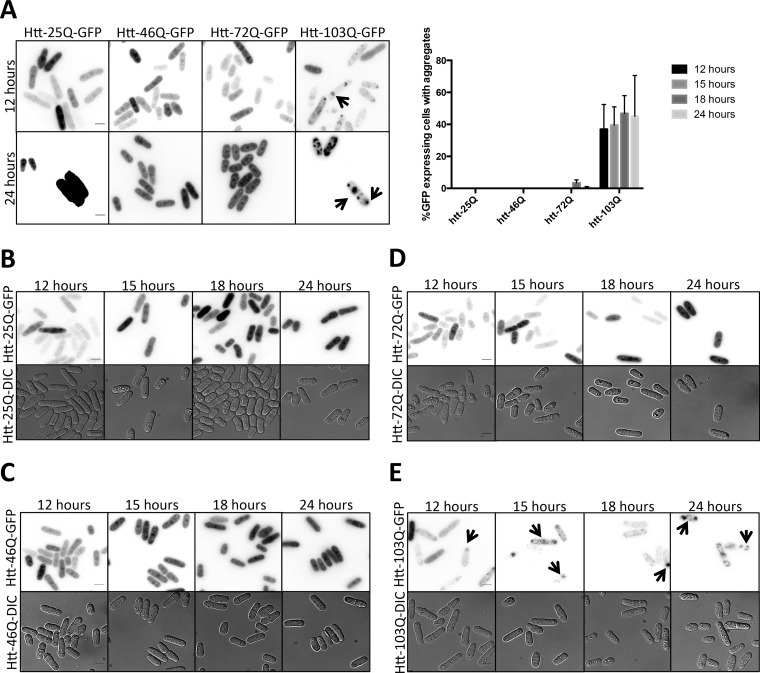
Aggregation of long polyQ-containing Htt proteins expressed in *S. pombe*. (A) Left, live-cell GFP microscopy of *htt*-25Q-, *htt*-46Q-, *htt*-72Q-, and *htt*-103Q-integrated *S. pombe* after 12 h (top) and 24 h (bottom) of induced expression. Images are presented with similar levels of contrast. 25Q is soluble and very highly expressed, leading to saturated intensity. Right, quantification of cells containing aggregated proteins per cells expressing GFP represented as the means of the results of three experiments ± standard errors of the means (SEM). (B to E) Time course of induction of *S. pombe* expressing (B) Htt-25Q-GFP, (C) Htt-46Q-GFP, (D) Htt-72Q-GFP, and (E) Htt-103Q-GFP. DIC, differential inference contrast. Scale bar, 5 µm.

To study the aggregation of various polyQ Htt constructs in *S. pombe*, we first performed fluorescence microscopy to visualize GFP (Fig. 1). For the 25Q and 46Q constructs, the fluorescence signal was uniformly and evenly distributed throughout the cytoplasm and nucleus (Fig. 1A to C). The fluorescence associated with Htt-72Q was also generally distributed evenly throughout the cytoplasm, with occasional small aggregates also arising (Fig. 1A and D). Expression of 103Q led to the presence of both small and large aggregates throughout these cells (Fig. 1A and E, indicated by arrowheads). This aggregation was quantified by counting the number of GFP-expressing cells containing aggregates at various time points after induction. This analysis revealed that only 103Q-expressing cells (between 37.0% and 46.8% of the cells) consistently contained aggregates over a 24-h time course of protein induction, while very few 72Q-expressing cells (3.4% ± 1.9%) contained even a low number of aggregates after 18 h of expression (Fig. 1A, right). In contrast, 46Q- and 25Q-expressing cells contained no visible aggregates and instead always expressed diffuse GFP localization at all time points examined (Fig. 1A and C and Fig. 1A and B, respectively).

**FIG 2:**
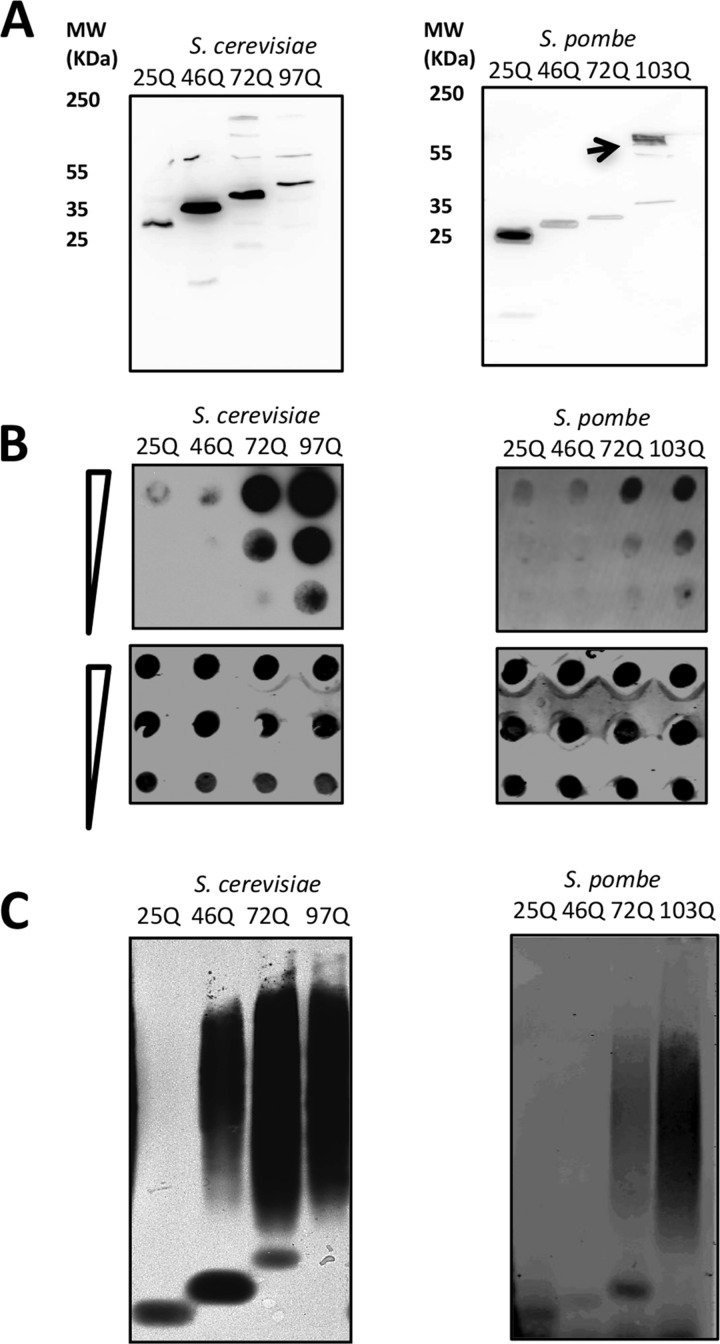
Detection of Htt protein aggregates in *S. cerevisiae* and *S. pombe*. (A) Left, Western blot analysis of *S. cerevisiae* htt-25Q, *htt*-46Q, *htt*-72Q, and *htt*-103Q using detection of an N-terminally fused FLAG tag with an arrow indicating a higher-molecular-mass aggregate; right, Western blot analysis of *S. pombe* htt-25Q, *htt*-46Q, *htt*-72Q, and *htt*-103Q using detection of an N-terminally fused FLAG tag. (B) Filter retardation assay of Htt-25Q-GFP, 46Q-GFP, 72Q-GFP, and 97Q-GFP expressed in *S. cerevisiae* (left) in 5-fold dilutions (top) and of the same strains with no SDS treatment (bottom) and filter retardation assay of Htt-25Q-GFP, 46Q-GFP, 72Q-GFP, and 103Q-GFP expressed in *S. pombe* (right) in 5-fold dilutions (top) and of the same strains with no SDS treatment (bottom). (C) SDD-AGE of *S. cerevisiae* expressing Htt-25Q-GFP, 46Q-GFP, 72Q-GFP, and 97Q-GFP (left) and of *S. pombe* expressing Htt-25Q-GFP, 46Q-GFP, 72Q-GFP, and 103Q-GFP (right) as detected by anti-FLAG antibody.

We next performed SDS-PAGE/Western blot and semidenaturating detergent agarose gel electrophoresis (SDD-AGE) assays to investigate Htt aggregation in *S. pombe* biochemically. SDS-PAGE/Western blot analysis revealed the presence of higher-molecular-mass aggregates for 103Q but not for 25Q, 46Q, and 72Q (Fig. 2A, indicated by arrowhead). Some higher-molecular-mass species are seen in the *S. cerevisiae* 72Q and 103Q lanes, and, additionally, the formation of many higher-molecular-mass aggregates likely made it impossible for them to enter the stacker gel for the 72Q and 103Q samples. We also performed SDD-AGE and filter retardation assays to analyze the formation of highly insoluble polyQ aggregates in *S. pombe* and to compare them to those in *S. cerevisiae*. Both assays showed that only 103Q in *S. pombe* produced insoluble protein aggregates with a higher molecular mass. In contrast, in *S. cerevisiae*, the accumulation of insoluble material began with 46Q and became even more pronounced for 72Q, consistent with previous studies (27, 28). These results show that longer polyQ expansions are required for aggregation in *S. pombe* versus *S. cerevisiae*. In addition, the cellular environment of *S. pombe* protects against aggregation of Htt constructs such as 46Q and 72Q, which aggregate and are associated with HD in humans.

### Expanded Htt does not cause a growth defect in *S. pombe*.

**FIG 3:**
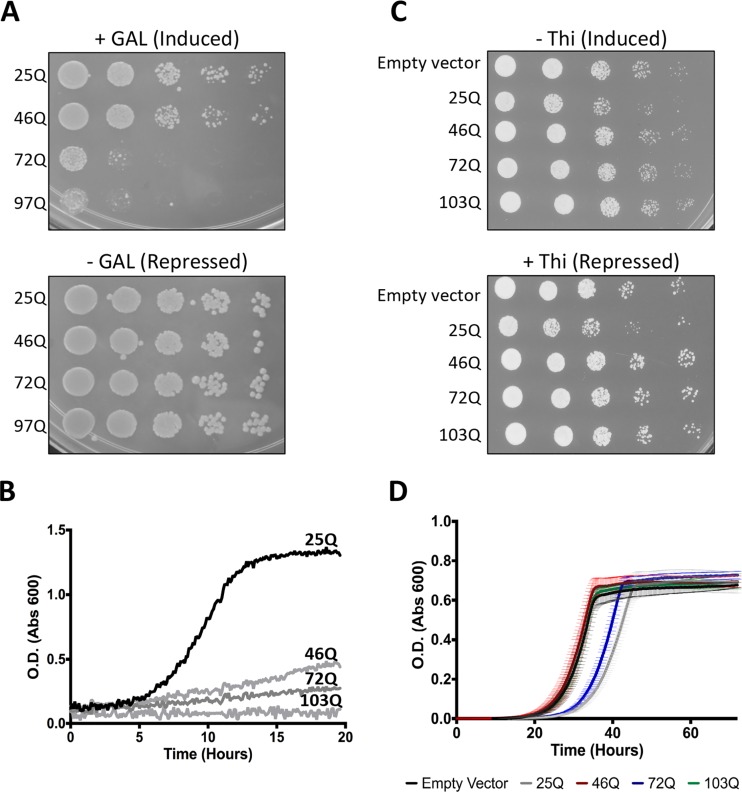
Effect of Htt on growth of *Saccharomyces cerevisiae* and *Schizosaccharomyces pombe*. (A) Htt exon 1 with various lengths of CAG repeats (25, 46, 72, and 97 bp) was integrated into *S. cerevisiae*, and cells were serially diluted 5-fold and spotted onto repressing (glucose [−GAL]) or inducing (galactose [+GAL]) solid media, as well as onto solid rich media. (B) The same *S. cerevisiae* strains expressing Htt exon 1 with various polyQ repeats were assayed in liquid minimal media by measuring the OD_600_ every 10 min. Abs, absorption. (C) Htt exon 1 with various lengths of CAG repeats was integrated into *S. pombe*, and cells were serially diluted 5-fold and spotted onto repressing (with thiamine [+Thi]) or inducing (without thiamine [−Thi]) solid media. (D) The same *S. pombe* strains were also assayed in liquid minimal media by measuring the OD_600_ every 15 min for 72 h. Average mean values ± SEM from triplicate measurements for each sample are shown.

We next investigated the effects of polyQ Htt expression on growth in yeast cells. It has been previously reported that expression of 72Q and, to a lesser extent, 46Q but not expression of 25Q causes a growth defect in *S. cerevisiae* (27). We reproduced this result on solid media (Fig. 3A) and also performed a liquid growth assay measuring the optical density at 600 nm (OD_600_) over a 20-h growth period after induction. The liquid growth assay clarified the mild growth defect for *S. cerevisiae* cells expressing 46Q (Fig. 3C). Taken together, these results confirm that the growth of *S. cerevisiae* cells is inhibited by expression of polyQ-expanded Htt in a polyQ length-dependent manner that starts with 46Q.

In contrast, when *S. pombe* cells expressing Htt-25Q, Htt-46Q, Htt-72Q, and Htt-103Q were spotted on solid media, none of the strains exhibited a significant degree of defective growth (Fig. 3B). Similarly, the results of a liquid assay monitoring OD_600_ over a 72-h growth period showed no significant growth defect for *S. pombe* strains expressing Htt-46Q, Htt-72Q, or Htt-103Q (Fig. 3D; see also Fig. S3 in the supplemental material). Consistent with these population-based growth assays, we observed no morphological signs of cellular toxicity in individual *S. pombe* cells containing Htt-103Q aggregates at the microscopic level (Fig. 1A and E). We also did not observe a growth defect upon expression of Sup35p and Ure2p in fission yeast cells. These aggregation-prone proteins are the protein determinants of the *S. cerevisiae* prions [PSI+] and [URE3] (see Fig. S4). Taken together, our data indicate that *S. pombe* cells display a marked resistance to both the aggregation and the toxicity of extended Htt polyQ proteins.

### Heat shock increases Htt aggregation but fails to induce a growth defect in *S. pombe.*

**FIG 4:**
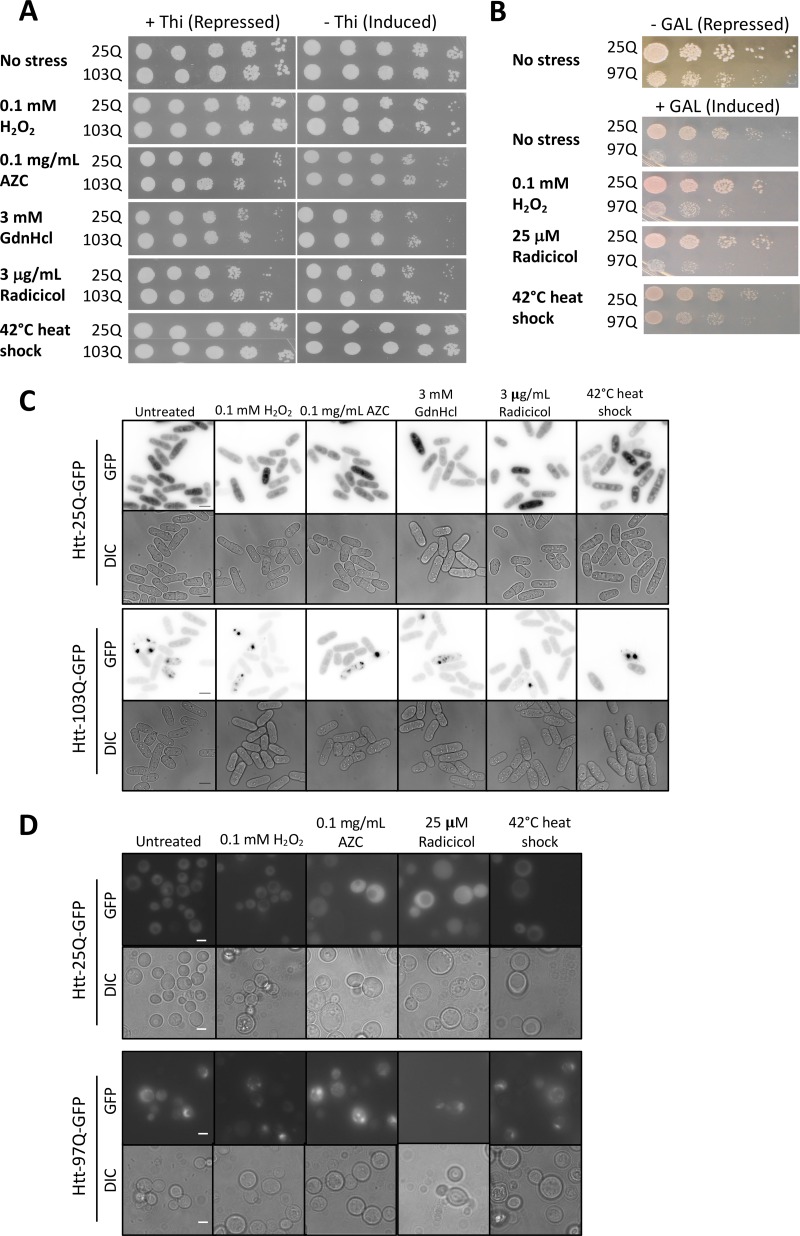
Chemical and heat stress of integrated Htt exon 1-expressing *Schizosaccharomyces pombe* and *Saccharomyces cerevisiae* strains with various lengths of CAG repeats. (A) Spotting assay of *S. pombe* htt-25Q and *htt*-103Q strains subjected to various stressors, as indicated. (B) Spotting assay of *S. cerevisiae* htt-25Q and *htt*-103Q subjected to various stressors, as indicated. (C) Live GFP microscopy of *S. pombe* htt-25Q (top) and *htt*-103Q (bottom) after Htt induction and 1 h of incubation in H_2_O_2_, l-azetidine-2-carboxylic acid (AZC), guanidine hydrochloride (GdnHCl), or radicicol or after 42°C heat shock for 1 h. Scale bar, 5 µm. (D) Live GFP microscopy of *S. cerevisiae* after Htt induction and 1-h incubation in H_2_O_2_, l-azetidine-2-carboxylic acid, guanidine hydrochloride, or radicicol or after 42°C heat shock for 1 h. Scale bar, 5 µm.

It has been shown that subjecting *D. discoideum* cells expressing Htt-103Q to heat stress caused a growth defect and the accumulation of aggregates not seen at normal temperature (29). Therefore, we tested whether any stress conditions might induce aggregation and/or a growth defect(s) in *S. pombe* expressing Htt-103Q. Heat shock applied for 1 h at 42°C increased the percentage of *S. pombe* cells with Htt-103Q aggregates (38% compared with 15% in untreated cells, *P* < 0.05 by test of proportions) (Fig. 4C), and *S. cerevisiae* showed results similar to those previously described for unstressed conditions (27). In contrast, no significant differences in the percentages of cells with aggregates were observed following treatment with H_2_O_2_, l-azetidine-2-carboxylic acid (AZC), guanidine hydrochloride, or radicicol in either *S. pombe* or *S. cerevisiae* (~14% to 25%, *P* > 0.05 by test of proportions) (Fig. 4C and D). Interestingly, none of these stress conditions caused either a growth defect (Fig. 4A) or a change in cellular morphology in *S. pombe* (Fig. 4C) or *S. cerevisiae* (Fig. 4B and D). For the *S. cerevisiae* samples, no growth was seen for either the Htt*-*25Q or the Htt*-*103Q samples after treatment with 0.1 mg/ml AZC following replating for spotting assays, making comparisons between strains impossible. Additionally, treatment with guanidine hydrochloride was not included for these samples as it has previously been shown that guanidine hydrochloride will cure these cells of their RNQ^+^ state, which has been shown to be required for toxicity (30).

### Partial nuclear localization of Htt-103Q aggregates in *S. pombe.*

**FIG 5:**
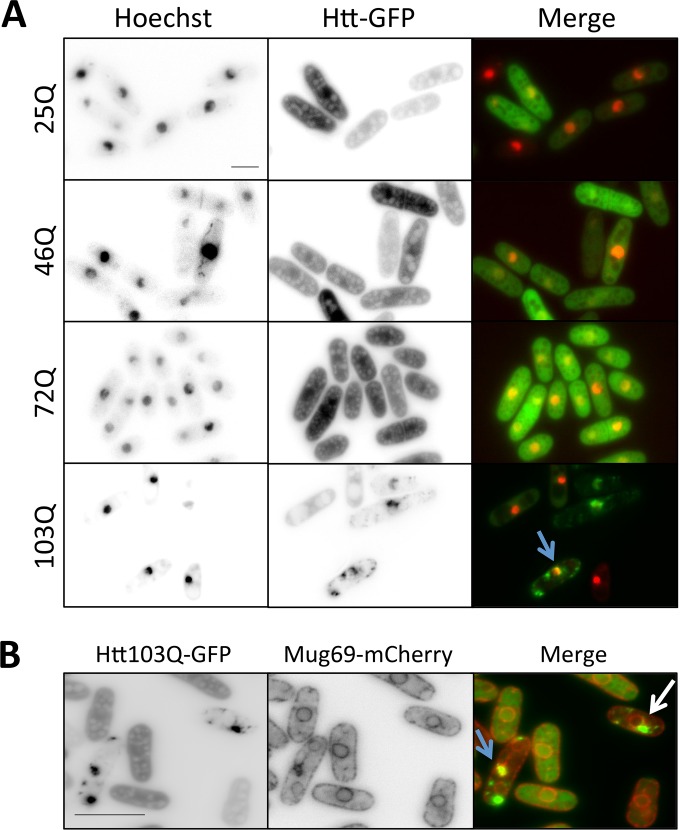
(A) Subcellular localization of Htt aggregates in *S. pombe*. Results of live-cell microscopy of Htt-polyQ constructs are shown in the middle panels, corresponding Hoechst-stained images in the left panels, and merged images in the right panels. (B) Left, microscopy of *htt-*103Q-GFP; in the center, endogenously tagged *mug69*-mCherry; on the right, a merged image. Scale bar, 5 µm.

Nuclear localization of polyQ Htt aggregates is observed in the neurons of HD patients, as well as in those examined in a variety of animal and cellular models (31–39). Notably, forced nuclear targeting of Htt-polyQ dramatically increases toxicity in *S. cerevisiae* (40, 41) and accelerates neurodegeneration in a mouse model (42), suggesting that nuclear localization is critical for polyQ-induced toxicity. To characterize the cellular localization of Htt aggregates in *S. pombe*, we used Hoechst staining to label the nuclei of cells expressing Htt-25Q, 46Q, 72Q, and 103Q and used fluorescence microscopy to visualize Htt localization relative to the nucleus. The results show that the majority of 103Q aggregates localized to the cytoplasm (Fig. 5A) but that a subset of aggregates localized to the nucleus (~12% [arrow]). These results argue that the lack of a growth defect in *S. pombe* cells expressing Htt-103Q cannot be attributed to the lack of nuclear aggregates.

### Inefficient sequestration of short endogenous polyQ proteins into Htt-103Q aggregates.

We next tested whether endogenous polyQ expansion proteins colocalize with Htt-103Q aggregates in *S. pombe. S. pombe* contains only three endogenous proteins (Mug69, Med15, and Sol1) expressing 10-Q stretches, which is the minimal threshold defining polyQ regions (2). For these experiments, we used an endogenously mCherry-tagged version of *S. pombe* polyQ protein Mug69 and coexpressed it with Htt-103Q GFP in these cells. We found that Htt-103Q aggregates did not efficiently recruit and sequester Mug69-mCherry (Fig. 5B, blue arrow). Rather, only a fraction (~25%) (Fig. 5B, white arrow) of strong Htt-103Q aggregates showed a concentration of Mug69-mCherry, meaning that Mug69 localization was largely unaffected by polyQ-containing aggregates. These results indicate that Htt-103Q does not efficiently sequester *S. pombe* polyQ proteins into aggregates.

### **CAG**/**CAA** regions are genetically stable in *S. pombe.*

**FIG 6:**
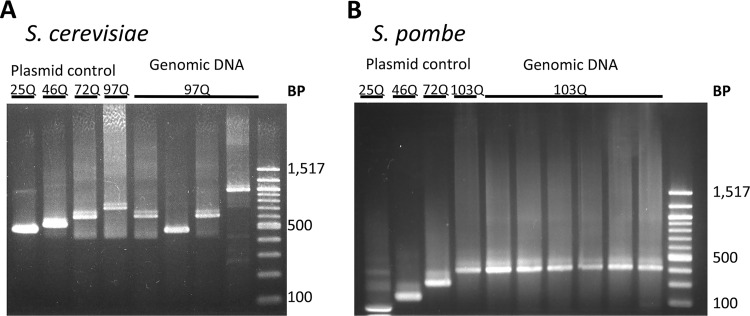
Genetic stability of Htt polyQ repeats in yeast. Genomic DNA was prepared from liquid cultures of six individual transformants of (A) *S. cerevisiae* and (B) *S. pombe*, and PCR analysis amplifying the region between the FLAG tag and the GFP tag was performed to look at the length of the CAG/CAA fragments. The data are compared to results of PCR analysis of the same fragments for the corresponding plasmid used for integration.

Long stretches of trinucleotide repeats in DNA are predisposed to lengthening in human germ line cells, causing a phenomenon known as anticipation in the inheritance of HD, whereby successive generations of patients in a pedigree exhibit earlier onset of disease with more-severe symptoms (43, 44). It has previously been reported that long trinucleotide repeat regions are also genetically unstable when integrated into *S. cerevisiae* cells, possibly due to the action of *RAD27*, which is involved in long-patch excision and other DNA repair processes (45). We therefore examined the genetic stability of expanded CAG/CAA mixed repeat regions encoding polyQ expansions in our Htt constructs expressed in *S. pombe*, an organism that lacks endogenous long CAG repeat domains. We prepared genomic DNA from isolated individual colonies of *S. pombe* and *S. cerevisiae* cells transformed by integration of DNA encoding Htt-103Q and measured the CAG/CAA repeat length by PCR with primers that included the CAG/CAA repeat domain followed by agarose gel electrophoresis. Four independent *S. cerevisiae* transformants produced PCR products of various lengths (Fig. 6A, lanes 5 to 8), none of which was the exact expected length, as indicated by the PCR product of the original Htt-103Q construct on plasmid DNA (Fig. 6A, lane 4). In contrast, PCR amplification of genomic DNA from each of six independent *S. pombe* transformants produced one single band of a length identical to that seen with the PCR product of the original construct on plasmid DNA (Fig. 6B, lanes 5 to 10 compared to lane 4). (Note that the absolute molecular masses of the plasmid control PCR products differ between *S. cerevisiae* and *S. pombe* due to need to use different primers for the two species.) We conclude that long CAG stretches, genetically unstable in *S. cerevisiae*, are genetically stable in *S. pombe.*

## DISCUSSION

Huntington’s disease is a devastating neurological disease caused by the accumulation of the polyQ-containing protein huntingtin. The severity of the disease, as well as the age of onset, is affected by the length of the polyQ repeat (4, 5), making this feature of the disease a central focus of investigation.

More broadly, polyQ-containing proteins constitute a class of proteins that have normal biological functions, and the abundances of these proteins are differentially distributed across organisms. PolyQ proteins are known for their ability to mediate protein-protein interactions, both with other polyQ proteins and with other proteins in the cellular microenvironment (46). These interactions are critical for their biological function. For instance, *CLN3* RNA transcripts are clustered in *Ashbya gossypii* cells by polyQ-containing protein Whi3, which results in cyclin transcript distribution and asynchronous nuclear cycling (1). While the normal physiology of polyQ proteins has yet to be completely determined, increasing research points to their role in stabilizing protein interactions and activation of gene transcription (2). In *S. cerevisiae*, the functional sequestration by polyQ proteins has been shown to be extended to their interaction partners, where it has been demonstrated that Sup35 in its [PSI+]-aggregated state can sequester Sup45, a protein required for efficient translation termination (47). Furthermore, sequestration of polyN proteins has also been demonstrated in *S. cerevisiae*, where [SWI+] aggregates can sequester other asparagine-rich proteins such as the transcriptional activators of genes responsible for multicellularity (48). In addition, Q/N-rich proteins may also play a role in the pathogenesis of neurological diseases (49), such as ALS and frontotemporal lobar degeneration with ubiquitin-only-immunoreactive neuronal changes (FTLD-U). It has become clear that altering the normal protein-protein interactions of aggregation-prone proteins, including polyQ Htt, dramatically influences their neuropathological effects (8, 9). This raises two questions: (1) how do polyQ-mediated protein-protein interactions determine the regular function of polyQ proteins, and (2) how does polyQ expansion cause disease in different cell types with different levels of aggregation-prone proteins and different protein quality control systems?

Recently, Malinovska et al. (26) explored the effect of expressing Htt-103Q in *Dictyostelium discoideum*, an organism with more than 10% of its proteome consisting of polyQ proteins (2) and an additional 13% of its proteome consisting of proteins with Q/N-rich aggregation-prone prion domains (26). That study found little toxicity and aggregation under normal growth conditions; however, under conditions of heat stress, the cells accumulated insoluble protein as their proteostatic machinery became compromised and died. These interesting results imply that polyQ-containing proteins are relatively benign in environments that have evolved to provide an adequate protein quality control system.

*S. pombe* provides us with a unique way to investigate this possibility from the opposite perspective, i.e., in a cellular environment with only three polyQ proteins expressed and with only one 10-Q region in each protein. Here, we found that heterologous expression of expanded polyQ Htt showed only aggregates of the longest polyQ expansion and never produced polyQ toxicity. Notably, these polyQ aggregates were sometimes found in the *S. pombe* nucleus, suggesting that even partial nuclear localization does not suffice to produce polyQ toxicity in *S. pombe*. Instead, it appears that polyQ Htt aggregates do not target other *S. pombe* proteins for sequestration. The most likely explanation for this observation is that, in cells with essential endogenous polyQ proteins, pathogenic polyQ aggregates sequester the endogenous polyQ proteins into a functionally inactive or inaccessible state, as has been previously found in organisms that endogenously contain proteins with polyQ stretches of more than 10, including CREB-binding protein and TATA-binding protein (10–15, 39). A hint of this type of sequestration mediated by interactions between different polyQ domains can be seen in our observation that Mug69 moderately coaggregated with Htt-103Q. However, the rarity of this colocalization suggests that it is a relatively weak interaction, most likely because the short length of the Mug69 polyQ region reduces its ability to self-aggregate. Thus, the majority of Mug69 remains normally localized and functional, and the cell exhibits no growth defect. Additionally, stressing *S. pombe* heterologously expressing Htt-103Q results in no change in toxicity or aggregate formation for a variety of chemical stressors, and the number of Htt-103Q aggregates increases only under conditions of heat stress. Unlike in *D. discoideum*, however, this does not result in toxicity (26), which further emphasizes the finding that Htt-103Q aggregation in *S. pombe* does not lead to cellular toxicity. In *S. cerevisiae*, it appears that Htt-103Q demonstrates the maximum amount of toxicity in the cell’s normal environment and that adding stressors therefore has little additive effect. By comparing these diverse organisms, our work suggests that endogenous polyQ repeats of minimal length have the potential to confer resistance to polyQ-driven aggregation and toxicity.

The inherent genetic instability of trinucleotide repeat regions is well documented in *S. cerevisiae*, as well as in human HD patients, where polyQ lengthening can lead to anticipation (43–45). Looking at the CAG/CAA repeat regions in *S. pombe*, however, we find that even the longest polyQ-encoding region, consisting of 103 CAG/CAA repeats, is genetically stable. This suggests that *S. pombe* may lack a molecular mechanism to allow changes in trinucleotide repeat region length, which in turn may provide an explanation for the lack of long polyQ repeats in its proteome.

Taken together, our data suggest that 103Q is nontoxic for different reasons in *S. pombe* versus *D. discoideum*. *D. discoideum* possesses a strong protein quality control system that prevents polyQ aggregation and toxicity, at least under normal growth conditions. In contrast, *S. pombe* does not prevent polyQ aggregation but also has no essential endogenous polyQ proteins that are sequestered into polyQ aggregates to render polyQ aggregation toxic. Our model is also consistent with prior work from the Gitler group, who showed that an endogenous polyQ protein (ataxin-2) that has only a moderately expanded polyQ tract can influence host cell susceptibility to the toxic protein TDP-43 (16, 18). An alternative but not mutually exclusive interpretation is that distinct protein quality control systems in different cells may prevent the formation of toxic oligomers and may instead favor the formation of either benign polyQ aggregates or soluble polyQ species.

**FIG 7:**
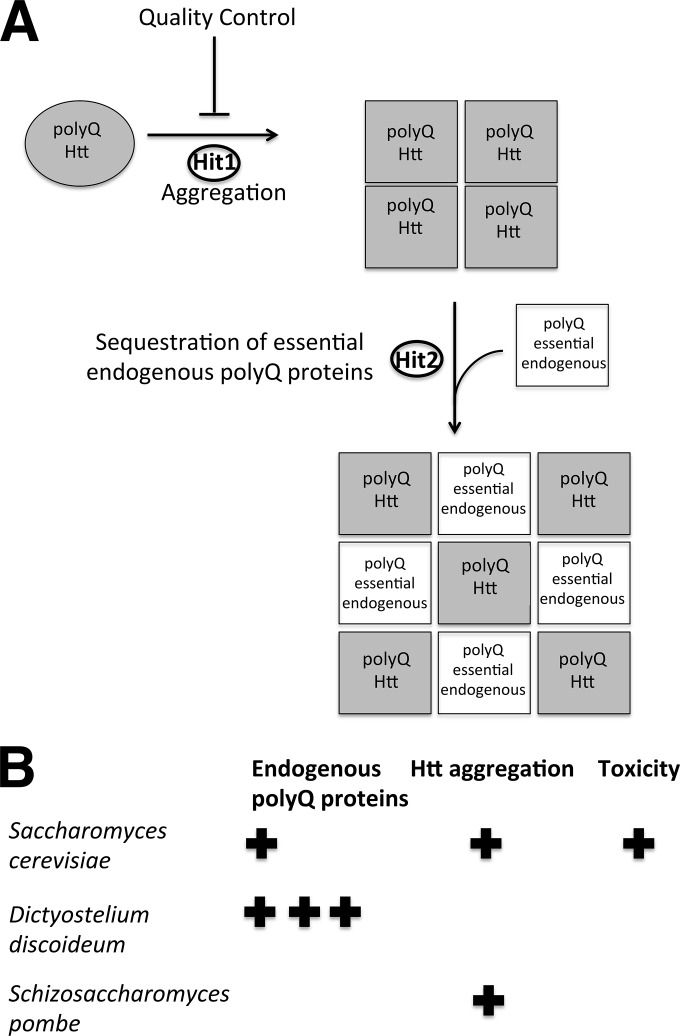
(A) Proposed two-hit model of Htt aggregation and toxicity. Htt expressed without specialized protein clearance is toxic (Hit 1) and also contains targets for coaggregation such as endogenous polyQ proteins that have essential cellular functions (Hit 2). (B) Summary of results of experimental analyses of Htt aggregation and toxicity from *Saccharomyces cerevisiae*, *Dictyostelium discoideum*, and *Schizosaccharomyces pombe*.

We propose a two-hit model for polyQ expansion protein aggregation and toxicity governed by protein quality control and the cell’s content of other aggregation-prone proteins. In this model, polyQ expansion proteins are toxic when expressed in a cell in which they both generate aggregates (first hit) and then coaggregate with endogenous aggregation-prone proteins, thus disrupting essential cellular functions (second hit) (Fig. 7A). Such a process may occur in *S. cerevisiae* and in neurons affected by neurodegeneration in polyQ expansion diseases. In contrast, in an organism with a high content of endogenous aggregation-prone proteins, such as *D. discoideum* (26), these environments have evolved with protein quality control systems that effectively minimize aggregation and toxicity. Yet, when expressed in cells with very few endogenous aggregation-prone proteins, such as *S. pombe*, polyQ expansion proteins ineffectively aggregate and are nontoxic because no endogenous aggregation-prone proteins are sequestered or trigger the production of toxic conformers of polyQ expansion proteins. This two-hit model implies that specific endogenous polyQ proteins may be critical for polyQ expansion-induced neurodegeneration and may thus explain the cell type-specific toxicity observed in polyQ expansion diseases.

## MATERIALS AND METHODS

### Yeast strains and methods.

*S. cerevisiae* strains were generated as previously described (27), under the expression of the *GAL1*-inducible promoter. Growth and induction for expression were performed by growing strains to mid-log phase in selective media containing a 1% glucose or 1% galactose carbon source and then pelleting and washing the cells 3 times with H_2_O before resuspending the cells in selective media with a 2% galactose or 0.2% glucose carbon source. Alternatively, cells were grown in selective media using a 2% glucose carbon source to mid-log phase followed by pelleting and washing the cells 3 times with H_2_O before resuspending in selective media with a 2% galactose carbon source.

To generate *S. pombe* constructs for genetic integration, DNA was amplified from *S. cerevisiae* plasmids using primers which flanked the sequence with an NheI restriction site on the forward primer and BamHI on the reverse primer (forward primer sequence, 5′-CGCGCGCTAGCATGGACTACAAGGAC-3′; reverse primer sequence, 5′-GCGCGGGATCCCCCGGGCTGCAGTT-3′). In the case of Ure2 and Sup35, DNA was amplified from *S. cerevisiae* plasmids (kindly provided by Aaron Gitler) using a NheI restriction site on the forward primer and BamHI on the reverse primer (Sup35 forward primer sequence, 5′-GATGCTAGCATGTGGATTCAAACCAA-3′; Sup35 reverse primer sequence, 5′-TGGGGATCCCTCGCAATTTTAACAAT-3′; Ure2 forward primer sequence, 5′-GATGCTAGCATGAGAATAACAACGGC-3′; Ure2 reverse primer sequence, tk;25′-TGGGGATCCTTCACCACGCAATGC-3′). pJM459, pJM460, and pJM457 (pJK148 backbone and C-terminal monomeric enhanced GFP [mEGFP] with P3nmt1, P41nmt, and P81nmt1 promoters, respectively) were digested with the respective enzymes (New England Biolabs, Ipswich, MA), and the digested vectors were gel purified after electrophoresis in 1% agarose (Promega, Madison, WI) dissolved in 1× Tris-acetate-EDTA (TAE) buffer. Ligation of constructs was done using T4 DNA ligase (New England Biolabs).

*S. pombe* strains and media were made using standard methods (50), transforming into JM837 (leu1-32 h-). Strains are listed in Table S1 in the supplemental material. For genetic tagging, PCR and homologous recombination were performed as previously described (51). Growth and induction of strains were performed in selective or minimal media containing 15 µM thiamine (Sigma Aldrich, St. Louis, MO) to mid-log phase, followed by pelleting and washing the cells 3 times with H_2_O before resuspending in selective or minimal media without thiamine.

### Microscopy.

All microscopy was done as previously described (52) using a Deltavision imaging system (Applied Precision, Issaquah, WA) constituting a customized Olympus IX-71 inverted wide-field microscope, a Photometrics CoolSNAPHQ2 camera, and an Insight solid-state illumination unit. Cells were imaged in liquid media under a coverslip using a single focal plane, and the resulting images were analyzed in ImageJ (National Institutes of Health).

### Spotting and liquid assay.

Spotting and liquid assays for both *S. pombe* and *S. cerevisiae* were done as previously described (27, 53). Briefly, the spotting assay was performed with induced cells by determining OD_600_ and diluting cells to equal concentrations by spotting five serial 5-fold dilutions starting with the most concentrated spot, containing approximately 100,000 cells. Spotting was done on rich media, selective media with the respective promoter repressed, and/or selective media with the selective promoter induced. The liquid assay for *S. cerevisiae* strains was performed using a Bioscreen C instrument measuring OD_600_ every 10 min; the liquid assay for *S. pombe* strains was done using a Molecular Devices Filter MaxF5 Multi-Mode Microplate Reader (Sunnyvale, CA) instrument measuring OD_600_ every 15 min.

### Hoeschst staining.

Hoeschst 3342 (Sigma Aldrich) was incubated with cells at a working concentration of 50 µg/ml for 5 min before pelleting and resuspending for microscopy were performed.

### Genomic DNA and PCR.

Five milliliters of saturated rich medium culture was spun down and resuspended in 1 ml of H_2_O and transferred to a screw top tube and spun down again. The pellet was resuspended in 200 µl lysis buffer (2% Triton X-100, 1% SDS, 100 mM NaCl, 10 mM Tris [pH 8], 1 mM EDTA) with 200 µl 25:24:1 phenol-chloroform/isoamyl alcohol and 200 mg glass beads. Samples were lysed using a Mini-BeadBeater 16 instrument (Biospec, Bartlesville, OK) at 4°C for 3 to 5 min and briefly spun down to remove the phenol from the lid. Two hundred microliters of 1× Tris-EDTA (TE) was added, and samples were inverted for mixing and then centrifuged for 5 min at maximum speed. The top layer was transferred to a new tube, and 1 ml of room temperature (RT) 100% ethanol was added. Samples were centrifuged at maximum speed for 2 min, decanted, and washed with 1 ml cold 70% ethanol. After being spun for 2 min at maximum speed, the supernatant was removed and the resulting pellet was allowed to dry at room temperature.

PCR was done using primers upstream from the GAL promoter for the forward direction and in the CFP tag downstream from the CAG/CAA-repeat region in the reverse direction (forward primer sequence, 5′-GGGGTAATTAATCAGCGAAGCG-3′, reverse primer sequence, 5′-GCATCACCTTCACCCTCTCCAC-3′) for *S. cerevisiae* samples. For *S. pombe* samples, PCR was done using same primers used for cloning, amplifying from the FLAG tag in the forward direction (forward primer sequence, 5′-CGCGCGCTAGCATGGACTACAAGGAC-3′) and after the CAG/CAA region in the reverse direction (reverse primer sequence, 5′-GCGCGGGATCCCCCGGGCTGCAGTT-3′). Electrophoresis was performed using 2% agarose (Promega) dissolved in 1× TAE buffer, and samples were stained with SYBR gold (Life Technologies, Carlsbad, CA).

### Htt detection.

To detect Htt protein expression, 2 OD_600_ units of cells were collected by measuring OD_600_ during induction in mid-log phase. The protein from harvest cells was purified by resuspending pelleted cells in 300 µl of trichloroacetic (TCA) buffer (10 mM Tris [HCl pH 8.0], 10% trichloroacetic acid, 25 mM NH_4_OAc, 1 mM Na_2_EDTA) followed by addition of a half-volume of glass beads. A Mini-BeadBeater 16 instrument was used at 4°C with two consecutive 1-min bursts, keeping the tubes chilled on ice between the bursts. Cell lysate was then transferred to a new microcentrifuge tube on ice, and cells were centrifuged for 10 min at 16,000 × *g* at 4°C to pellet the precipitated proteins and cell debris. The supernatant was removed by aspiration and the pellet resuspended in 150 µl of resuspension solution (0.1 M Tris-HCl [pH 11.0], 3% SDS) and boiled for 5 min before being allowed to cool to room temperature. Samples were then centrifuged for 30 s at 16,000 × *g* to pellet cell debris, and the supernatant containing protein samples was reserved and assayed for protein concentrations using the BioRad DC protein assay (BioRad, Hercules, CA). Proteins were separated using SDS-PAGE and transferred to a polyvinylidene difluoride (PVDF) membrane as previously described (54) followed by Western blot detection using a monoclonal anti-FLAG antibody (Sigma, St. Louis, MO) and secondary detection using horseradish peroxidase-labeled anti-mouse IgG conjugate (GE Healthcare). Immunoblots were developed using West Femto (Pierce, Rockford, IL) chemiluminescence substrate and digitally documented using a Fuji (Fujifilm, Tokyo, Japan) LAS-3000 system.

### Stress condition experiments.

Heat stress of *S. pombe* strains was performed by initially inducing protein expression by thiamine withdrawal for 18 h at 30°C and then placing cells in a 42°C shaking water bath for 1 h prior to spotting assays and microscopy.

H_2_O_2_ toxicity and l-azetidine-2-carboxylic acid, guanidine hydrochloride, and radicicol toxicity experiments were done by incorporating the respective agents into minimal media at the given concentrations and as previously described for selective media (55–57) and by performing spotting assays as described above. For microscopy, cells were induced for protein expression by thiamine withdrawal for 16 h at 32°C (*S. pombe*) or growth in 2% galactose or 0.2% glucose at 30°C and then pelleted by centrifugation and resuspended in treated media containing the respective agents at the given concentrations.

### Filter retardation assay.

The filter retardation assay was done as previously described (58). Briefly, samples were diluted 5-fold in phosphate-buffered saline (PBS) or in PBS with 2% SDS. Samples were then absorbed onto a 0.2-μm-pore-size nitrocellulose membrane (Protran Whatman, Sigma Aldrich) presoaked with PBS. The membrane was then blocked with milk and immunoblotted for polyQ-containing proteins.

### SDD-AGE.

Cells were grown to an OD_600_ of approximately 1.5 and washed once with H_2_O. Cells were then lysed in 300 µl lysis buffer (100 mM Tris [pH 7.5], 200 mM NaCl, 1 mM EDTA, 5% glycerol, 1 mM dithiothreitol [DTT], 5 µg/ml aprotinin, 5 µg/ml leupeptide, 8 mM phenylmethylsulfonyl fluoride [PMSF]) and 200 mg of glass beads using a Mini-BeadBeater 16 instrument (Biospec) at 4°C for 3 min. Supernatant was then removed to a fresh tube using a 20-gauge syringe to puncture the bottom of the tube and spinning for 2 min at 500 × *g*. Protein was then quantified using a Bradford assay. Gel was prepared by dissolving 1.8% agarose in 1× TAE, and after dissolving, adding SDS to reach a concentration of 2%. Samples were run in 1× sample buffer (2× TAE, 20% glycerol, 8% SDS, bromophenol blue) after incubation at RT for 10 min or at 95°C and run at 80 V for approximately 2 h. Transfer was done by rinsing the gel in H_2_O followed by Tris-buffered saline (TBS) (100 ml 100 mM Tris [pH 7.5], 9 g NaCl) and increasing the total volume to 1 liter with H_2_O, and the reaction mixture was blotted onto nitrocellulose using a TurboBlotter (GE Healthcare, Pittsburgh, PA) for 4 h.

## SUPPLEMENTAL MATERIAL

Figure S1  *S. pombe* Htt constructs. An N-terminal FLAG tag was fused to human huntingtin exon 1 followed by CAG repeats of various lengths (from the top, 25, 46, 72, and 103 bp) with a C-terminal GFP tag. Download Figure S1, TIF file, 0.6 MB

Figure S2  Western blot analysis using the FLAG epitope of Htt-46Q expressed in *S. cerevisiae* using a GAL1 promoter (left) and in *S. pombe* using the P41nmt1 promoter (“intermediate”) and P3nmt1 promoter (“strong”) (right). Protein expression was induced and monitored from liquid culture over the indicated hours of induction. Download Figure S2, TIF file, 0.1 MB

Figure S3  Two biological replicates of (A) *htt-*25Q, (B) *htt*-46Q, (C) *htt*-72Q, and (D) *htt*-103 *S. pombe* strains representing individual colonies were assayed in minimal media by measuring OD_600_ every 15 min for 72 h. Average mean values ± SEM from triplicate measurements for each sample are shown. Download Figure S3, TIF file, 0.2 MB

Figure S4  Ure2 and Sup35 were cloned into plasmids containing P81nmt1, P41nmt1, and P3nmt1 promoters and integrated into *S. pombe* strain JM837 (*leu1-32*). The resulting cells were serially diluted 5-fold and spotted onto repressing (with thiamine) or inducing (without thiamine) media. Download Figure S4, TIF file, 0.4 MB

Figure S5  Increased concentrations of chemical stressor applied to integrated Htt exon 1-expressing *Schizosaccharomyces pombe* htt-25Q and *htt-*103Q strains. (A) Live GFP microscopy of *S. pombe* htt-25Q and *htt*-103Q after 1 h of growth in H_2_O_2_, l-azetidine-2-carboxylic acid, or guanidine hydrochloride. Scale bar, 5 µm. Download Figure S5, TIF file, 1.4 MB

Table S1  List of yeast strains used in this study. Table S1, DOC file, 0.04 MB
