# Supplementary figures and images for: CAG Expansions Are Genetically Stable and Form Nontoxic Aggregates in Cells Lacking Endogenous Polyglutamine Proteins
Source: mBio. 2016 Sep 27;7(5):e01367-16. doi: 10.1128/mBio.01367-16 (PMC5040113; doi:10.1128/mBio.01367-16)

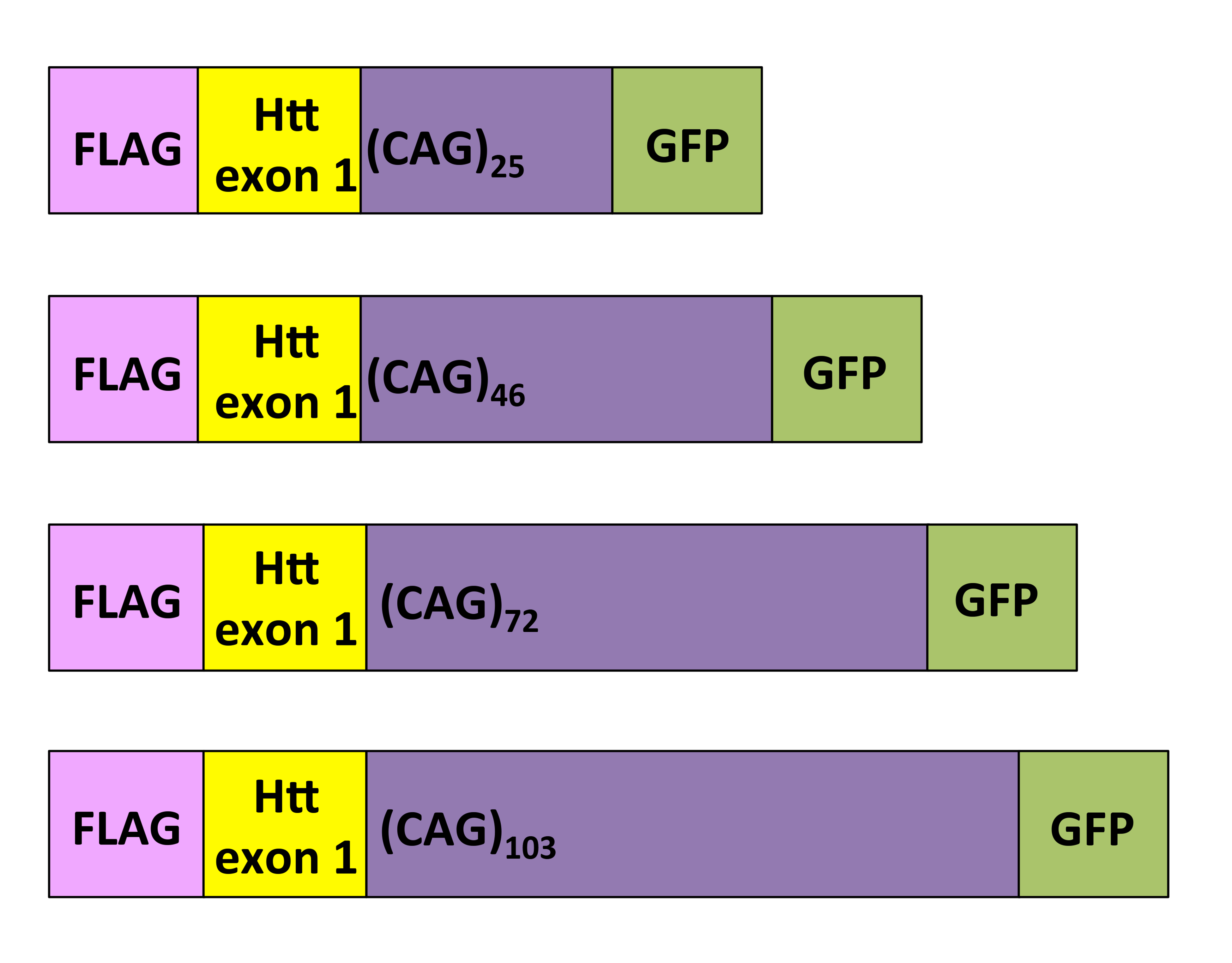

Supplement: Figure S1 — S. pombe Htt constructs. An N-terminal FLAG tag was fused to human huntingtin exon 1 followed by CAG repeats of various lengths (from the top, 25, 46, 72, and 103 bp) with a C-terminal GFP tag. Download [file mbo005163009sf1.tif]

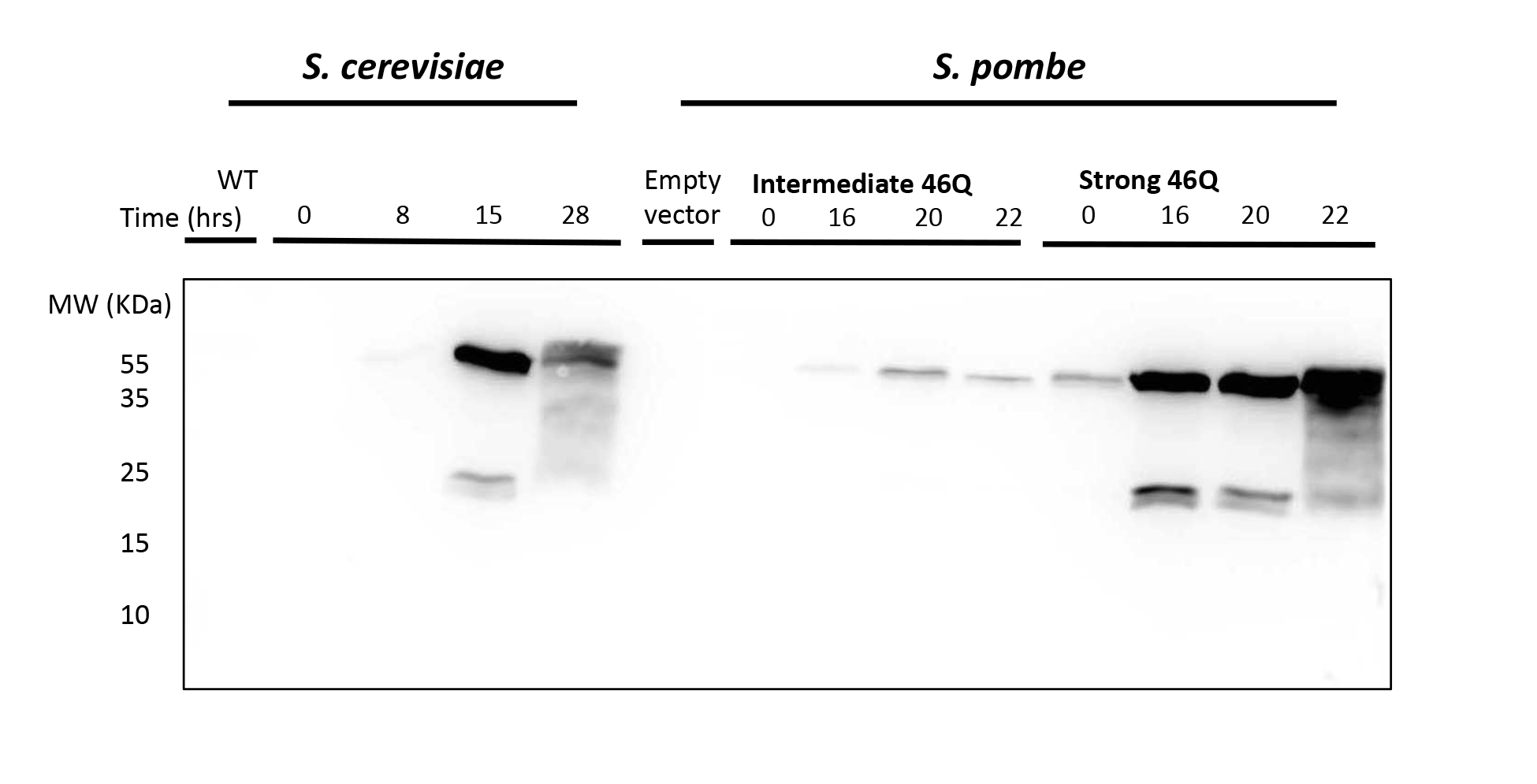

Supplement: Figure S2 — Western blot analysis using the FLAG epitope of Htt-46Q expressed in S. cerevisiae using a GAL1 promoter (left) and in S. pombe using the P41nmt1 promoter (“intermediate”) and P3nmt1 promoter (“strong”) (right). Protein expression was induced and monitored from liquid culture over the indicated hours of induction. Download [file mbo005163009sf2.tif]

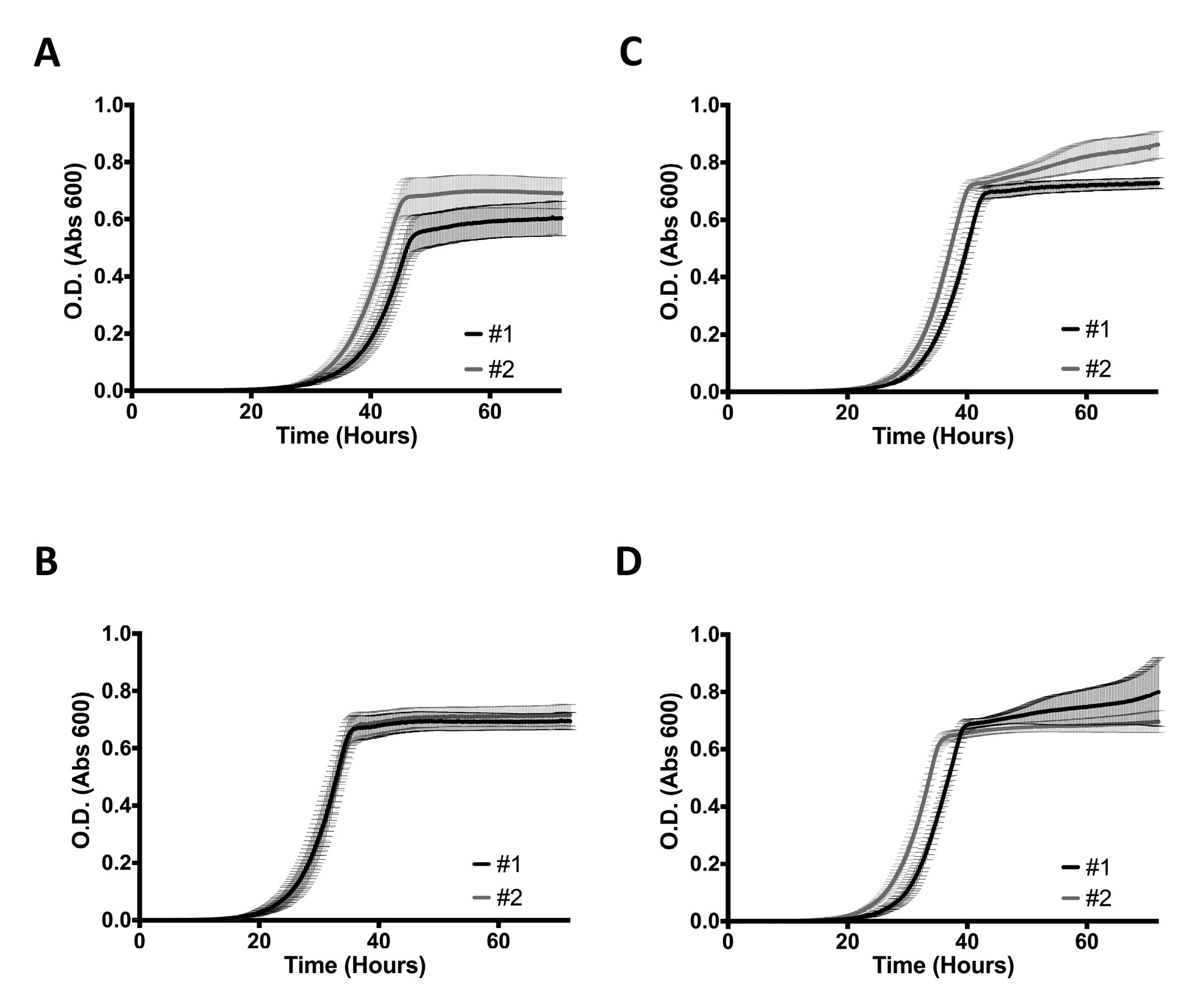

Supplement: Figure S3 — Two biological replicates of (A) htt-25Q, (B) htt-46Q, (C) htt-72Q, and (D) htt-103 S. pombe strains representing individual colonies were assayed in minimal media by measuring OD600 every 15 min for 72 h. Average mean values ± SEM from triplicate measurements for each sample are shown. Download [file mbo005163009sf3.tif]

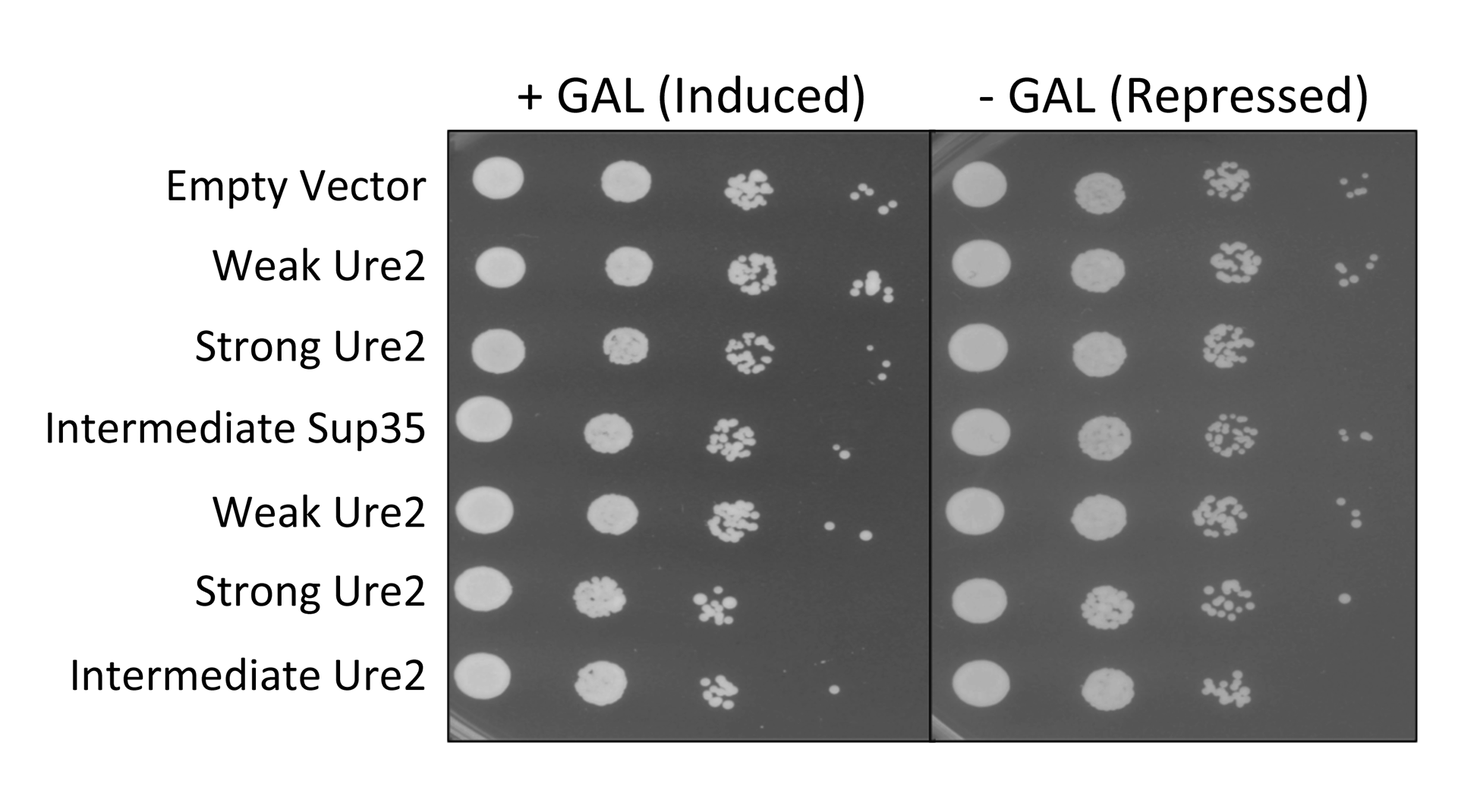

Supplement: Figure S4 — Ure2 and Sup35 were cloned into plasmids containing P81nmt1, P41nmt1, and P3nmt1 promoters and integrated into S. pombe strain JM837 (leu1-32). The resulting cells were serially diluted 5-fold and spotted onto repressing (with thiamine) or inducing (without thiamine) media. Download [file mbo005163009sf4.tif]

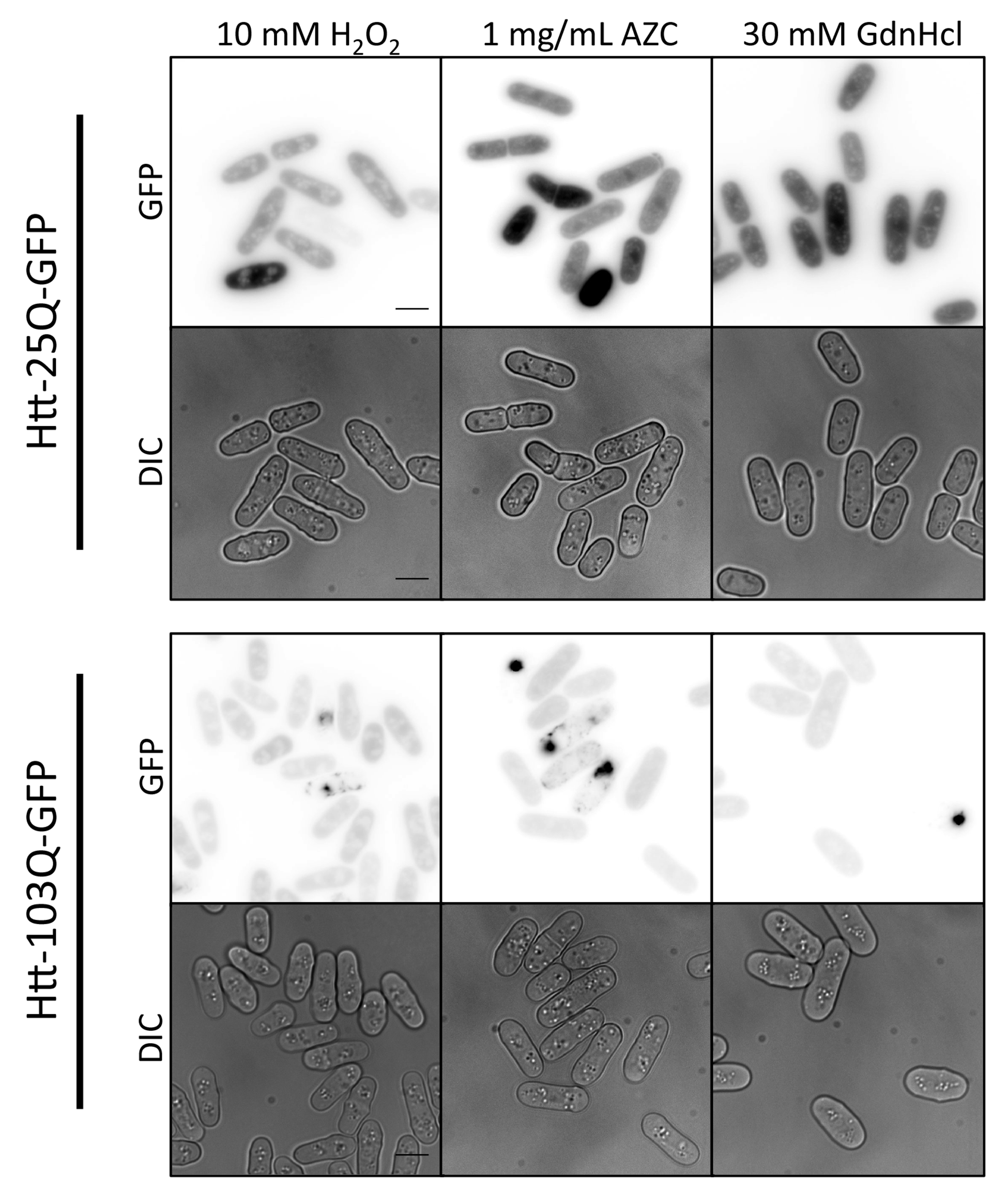

Supplement: Figure S5 — Increased concentrations of chemical stressor applied to integrated Htt exon 1-expressing Schizosaccharomyces pombe htt-25Q and htt-103Q strains. (A) Live GFP microscopy of S. pombe htt-25Q and htt-103Q after 1 h of growth in H2O2, l-azetidine-2-carboxylic acid, or guanidine hydrochloride. Scale bar, 5 µm. Download [file mbo005163009sf5.tif]
