# Supplementary material for: CAG Expansions Are Genetically Stable and Form Nontoxic Aggregates in Cells Lacking Endogenous Polyglutamine Proteins
Source: mBio. 2016 Sep 27;7(5):e01367-16. doi: 10.1128/mBio.01367-16 (PMC5040113; doi:10.1128/mBio.01367-16)
Supplement: Table S1 — List of yeast strains used in this study. [file mbo005163009st1.doc]

| **Table S1. Yeast strains used in this study** | |  |
| --- | --- | --- |
|  |  |  |
| **Strain Name** | **Genotype** | **Source** |
| JM837 | *leu1-32 h-* | Moseley Lab Collection |
| AZ001 | *P3nmt1-FLAG-HTT(25Q)-GFP::leu1+ leu1-32 h-* | This study |
| AZ002 | *P3nmt1-FLAG-HTT(46Q)-GFP::leu1+ leu1-32 h-* | This study |
| AZ003 | *P3nmt1-FLAG-HTT(72Q)-GFP::leu1+ leu1-32 h-* | This study |
| AZ004 | *P3nmt1-FLAG-HTT(103Q)-GFP::leu1+ leu1-32 h-* | This study |
| AZ006 | *P41nmt1-FLAG-HTT(46Q)-GFP::leu1+ leu1-32 h-* | This study |
| AZ013 | *P3nmt1-GFP::leu1+ leu1-32 h-* | This study |
| AZ014 | *P3nmt1-FLAG-HTT(103Q)-GFP::leu1+ Mug69-mCherry::KanMX6 leu1-32 h-* | This study |
| MD | *MATα PGAL1-FLAG-HTT(25Q)-CFP::his3+ can1-100 ade2-1 his3-11, 15 trp1-1 ura3-1 leu23,112* | Duennwald Lab Collection |
| MD | *MAT*α PGAL1-FLAG-HTT(46Q)-CFP::his3+ *can1*-*100 ade2*-*1 his3*-*11*, *15 trp1*-*1 ura3*-*1 leu23*,*112* | Duennwald Lab Collection |
| MD | *MATα PGAL1-FLAG-HTT(72Q)-CFP::his3+ can1-100 ade2-1 his3-11, 15 trp1-1 ura3-1 leu23,112* | Duennwald Lab Collection |
| MD | *MAT*α PGAL1-FLAG-HTT(103Q)-CFP::his3+ *can1*-*100 ade2*-*1 his3*-*11*, *15 trp1*-*1 ura3*-*1 leu23*,*112* | Duennwald Lab Collection |
